# Comparison of individual and pooled diagnostic examination strategies during the national mapping of soil-transmitted helminths and *Schistosoma mansoni* in Ethiopia

**DOI:** 10.1371/journal.pntd.0006723

**Published:** 2018-09-10

**Authors:** Gemechu Tadesse Leta, Mike French, Pierre Dorny, Jozef Vercruysse, Bruno Levecke

**Affiliations:** 1 Ethiopian Public Health Institute, Addis Ababa, Ethiopia; 2 Department of Virology, Parasitology and Immunology, Faculty of Veterinary Medicine, Ghent University, Merelbeke, Belgium; 3 RTI International, Washington D.C., United States of America; 4 Department of Biomedical Sciences, Institute of Tropical Medicine, Antwerp, Belgium; Centers for Disease Control and Prevention, UNITED STATES

## Abstract

**Background:**

Laboratory-based studies have highlighted that pooling stool and urine samples can reduce costs and diagnostic burden without a negative impact on the ability to estimate the intensity of soil-transmitted helminth (STH, *Ascaris lumbricoides*, *Trichuris trichiura* and hookworms) and schistosome infections (*Schistosoma mansoni* and *S*. *haematobium*). In this study, we compare individual and pooled stool examination strategies in a programmatic setting.

**Methods:**

Stool samples were collected from 2,650 children in 53 primary schools in Amhara Regional State, Ethiopia, during the national mapping of STHs and schistosome infections. Eggs of STHs and *S*. *mansoni* were quantified in both individual and pooled samples (pools were made from 10 individual samples) using a single Kato-Katz smear.

**Principal findings:**

A pooled diagnostic examination strategy provided comparable estimates of infection intensity with higher fecal egg count (expressed in eggs per gram of stool (EPG)) than those based on individual strategy (*Ascaris*: 45.1 EPG *vs*. 93.9, *p* = 0.03; *Trichuris*: 1.8 EPG *vs*. 2.1 EPG, *p = 0*.*95*; hookworms: 17.5 EPG *vs*. 28.5 EPG, *p = 0*.*18*; *S*. *mansoni*: 1.6 EPG *vs*. 3.4 EPG, *p* = 0.02), but had lower sensitivity (*Ascaris*: 90.0% *vs*. 55.0%; *Trichuris*: 91.7% *vs*. 16.7%; hookworms: 92.6% *vs*. 61.8%; *S*. *mansoni*: 100% *vs*. 51.7%, *p* < 0.001). A pooled approach resulted in a ~70% reduction in time required for sample testing, but reduced total operational costs by only ~11%.

**Conclusions/Significance:**

A pooled approach holds promise for the rapid assessment of intensity of helminth infections in a programmatic setting, but it is not major cost-saving strategy. Further investigation is required to determine when and how pooling can be utilized. Such work should also include validation of statistical methods to estimate prevalence based on pooling samples. Finally, the comparison of operational costs across different scenarios of national program management will help determine whether pooling is indeed worthwhile considering.

## Author summary

Infections with intestinal (roundworms, whipworm and hookworms) and blood-dwelling (schistosomes) worms pose a significant public health burden in developing tropical countries. To optimize control programs against these worms, large-scale surveys are required to determine the worm distribution to initiate control and to monitor the success of the programs. These large-scale surveys come at an important cost for governments, which in resource-limited countries present major challenges. During a nationwide survey in Ethiopia, we assessed whether the examination of pooled rather than individual samples could be a cost-saving strategy to assess prevalence and intensity of worm infections (which is an indicator of worm-related morbidity and success of the control program). We showed that a pooled examination strategy was useful in estimating the intensity of worm infections, but that it underestimated prevalence. Examination of pooled samples significantly reduced laboratory time, but it only resulted in limited financial gain.

## Introduction

Neglected tropical diseases (NTDs) are a group of parasitic, bacterial and viral infections that pose an important burden on public health, particularly in tropical and sub-tropical countries [1]. In 2015, this group of 17 diseases resulted in approximately 26 million disability-adjusted life years (DALYs) and are considered an issue of global importance hindering progress towards the Sustainable Development Goals [2]. Soil-transmitted helminthiasis (STH) and schistosomiasis (SCH) are two of seven NTDs that are amenable to control through regular mass drug administration (MDA) [3]. Millions of people are infected worldwide, with each disease attributable for more than 10% of the overall NTD burden (schistosomiasis: 11%; soil-transmitted helminthiasis: 14%) [4]. Both are targeted primarily through school-based treatment programs, during which anthelmintic drugs (albendazole or mebendazole for STH and praziquantel for SCH) are administered to all school age children [5]. Fueled by the London Declaration on NTDs (January 2012; [6]), the global treatment coverage of school-aged children has increased since 2011 for SCH (2011: <20% *vs*. 2016: 53.7%) and STH (2011: ~30% *vs*. 2016: 69.5% [7–9], with the ultimate goal of treating at least 75% of school-aged children in all endemic countries by 2020 [10].

The pharmaceutical industry donates anthelmintic drugs at-scale (albendazole: GlaxoSmithKline, mebendazole: Johnson & Johnson, praziquantel: Merck KGaA) [11]. However, MDA programs require substantial political and financial investments from endemic countries [12]. Additionally, there are costs for periodically assessing the epidemiology of the diseases. Prior to treatment, nationwide epidemiological surveys are used to target treatment appropriately. Periodic follow-up surveys are required to measure progress and determine whether scaling-down of MDA is justified [13].

Ethiopia published its first National Master Plan for NTDs in 2012 (2012–2015), outlining plans to scale-up MDA efforts for eight priority NTDs. For STH and SCH, Ethiopia mobilized financial resources through a range of partners to support its nationwide baseline surveys. Given the substantial cost of such surveys, there is a need to identify cost-effective mapping approaches to further drive country ownership. The examination of a pooled stool sample strategy (ten individual samples) rather than using individual samples is a potential cost-saving strategy. Evidence from veterinary medicine shows that pooling can reduce diagnostic burden and costs, without having a negative impact on estimating the intensity of helminth infections [14]. Pooling has been evaluated for the assessment of STH and SCH in humans [15,16], highlighting that a pooled approach holds promise for rapid assessment of infection, but lacks diagnostic sensitivity. However, previous studies have focused on a small-scale (number of samples: 116–840), confined geographical area (Ethiopia: Jimma Town [15,16] and Amibara District [17]; Côte d’ Ivoire: Azaguié health district [18]), moderate and high transmission areas (STH prevalence ~50% [15,16]; SCH prevalence ~25% [15,16] and ~50% [15,16]), and provided limited information on operational costs. In the current study, we compared an individual and pooled examination strategy for the detection and quantification of soil-transmitted helminth and *Schistosoma mansoni* infections in 2,650 children in 53 primary schools across 35 woredas of Amhara Regional State in Ethiopia. In addition, we compared the time for sample testing for a subset of the samples, and the total operational costs for both strategies.

## Methods

### Ethics statement

This study was embedded in the national mapping of STH (caused by *Ascaris lumbricoides*, *Trichuris trichiura* and the hookworms, *Necator americanus* and *Ancylostoma duodenale*) and SCH (caused by *S*. *mansoni* and *S*. *haematobium*) in Ethiopia. The study protocol was reviewed and approved by the Scientific and Ethical Review Office of the Ethiopian Public Health Institute (ref. no.: SERO-128-4-2005). The Regional Health and Education Bureau were informed about the survey. During the survey, school directors, teachers, and students were informed of its purpose, including its benefits, potential risks and operational procedures. Participation in the study was entirely voluntary. Written informed consent was obtained from the directors of all participating schools, and verbal consent was obtained from all subjects. Subjects who provided a stool sample were given a single dose of mebendazole 500 mg and subjects excreting eggs of *S*. *mansoni* or *S*. *haematobium* were provided a single dose of praziquantel (40 mg/kg of body weight).

### Study area and population

This study was conducted in Amhara Regional State in the North of Ethiopia (9° - 14° N and 36° - 40° E). Amhara consists of 10 zones and 157 woredas and is divided into three major ecological zones: the highlands (>2,300 m above sea level [asl]), midlands (1,500 to 2,300 m asl) and the lowlands (<1,500 m asl). The annual mean temperature is between 15°C and 21°C. The mean annual rainfall is 1,165 mm, with the highest rainfall from June to September. The region’s population is 17.2 million of which 2.4 million (14%) are 10 to 14 years [19].

### Qualitative and quantitative assessment of STH and *S*. *mansoni* infections

This study was part of a school-based cross-sectional study to map the distribution of STH and SCH in Amhara Regional State and was conducted from February to March 2015. Ten schools were randomly selected in each district. From this list, five schools were purposively selected based on (i) reports of schistosomiasis, (ii) the presence of water bodies close to the schools and (iii) practices of irrigation and fishing in the community. Finally, 50 grade-five pupils (9 to 14 years; 25 girls and 25 boys) were randomly selected. In schools with fewer than 25 boys or girls in the appropriate grades, children (9 to 14 years) from lower grades (grade four) or higher grade (grade six) were included.

Each subject was asked to provide a stool sample of approximately 3 g in order to examine samples individually and to subsequently make pools of 10 individual samples. All laboratory procedures were performed in the nearest District Health Facility. At the collection site, the stool samples were immediately stored in a cool box (at 4°C) to avoid development of hookworm eggs. On average, samples were kept in cool box for 2hrs prior to processing. At the District Health Facility, samples were processed individually using a single Kato-Katz thick smear, as described elsewhere [20]. Subsequently, stool samples were combined into pools of 10 samples. The procedure of pooling was based on the methodology described by Kure et al., 2015 [15] and is illustrated in S1 Fig. In summary, 50 individual samples per school were placed in 5 rows of 10 samples. From each individual, 1g of stool was transferred into a new pre-labeled stool cup and thoroughly mixed with a wooden spatula until the color of the mixture became uniform. Finally, the pools were processed applying a single Kato-Katz thick smear.

The sensitivity and specificity (based on faecal egg counts (FEC) expressed in eggs per gram of stool (EPG)) of the pooled examination strategy was determined. The sensitivity was calculated using the combined results of both strategies as the diagnostic ‘gold’ standard, against which the sensitivity of the different individual approaches were compared. Therefore, the specificity of both strategies was set at 100%, as indicated by the morphology of the eggs. The sensitivity was determined at the level of the pools and at the level of schools by comparing against the combined strategies. Differences in sensitivity between examination strategies were assessed by a permutation test taking into account the dependency of results within samples (10,000 iterations) [21]. The variation in sensitivity of a pooled examination over different levels of egg excretion was explored for each of the four helminths. The classification of the levels of egg excretion were based on the 33^th^ and 66^th^ quantile (q33 and q66) of the mean of the corresponding individual FECs, resulting in 3 levels of egg excretion (level 1: mean FECs ≤ q33; level 2: q33 < mean FECs ≤ q66; level 3: mean FECs > q66). Differences in sensitivity between levels was assessed by a permutation test taking into account the dependency of results within samples (5,000 iterations). Tukey’s method was applied for multiple comparisons [21]. The agreement between FEC obtained by examining a pooled sample with the mean FEC of the corresponding individual FECs was evaluated by a permutation test (5,000 iterations) based on Pearson correlation coefficient and differences in FEC. For the assessment of correlation, FECs of the pooled examination strategy and the mean FECs were log transformed. For the assessment of the difference, no transformation was applied.

### Time for sample testing

A comparison of time taken to prepare and examine a subset of the samples (n = 2,450) was conducted. The steps were (i) the preparation of Kato-Katz thick smears, (ii) the pooling of stool, and (iii) the examination of the Kato-Katz thick smear. Given that timing of the preparation for each individual Kato-Katz thick smear would slow down the workflow, we recorded the total time to make batches of 10 Kato-Katz thick smears. The preparation of pools and the examination of a Kato-Katz thick smear were recorded on an individual basis. The mean time and corresponding standard deviation was calculated for preparing and reading of the individual and pools across the five survey teams.

### Estimation of operational costs

The total operational cost to map soil-transmitted helminth and *S*. *mansoni* infections were estimated for both strategies. The operational costs were assumed to depend on (i) human resources utilised, (ii) the number of schools that could be screened in one day, and (iii) the time during which no activities linked to the survey could be performed. In the present study, five field teams were involved, each consisting of three laboratory technicians and one nurse supported by one vehicle. Our experience from previous surveys suggested that number of schools that can be screened by one team per day ranges from one to three depending on the schools’ accessibility. The teams obtained the permission of the Health and Education Office to operate within each district. Teams were on the road during the entire survey, but were not able to work over weekends as both schools and Health and Education Offices were closed.

We estimated the operational cost for one team to be on the road for twelve weeks across different scenarios of school accessibility. We first calculated the cost for one day of work at each school (e.g. driving to schools, sample collection and processing samples), administration (e.g. obtaining the permission to conduct the survey), travel, and days-off (a day without survey related activities) across the three levels of school accessibility. These costs included the expenses for materials, salary, transport, and fees to facilitate the work at the schools and data entry. The costs for the materials included equipment, supplies and reagents, based on an itemized cost assessment considering the cost per unit, the usage over a one year period, the life expectancy (in years), and the number of samples that can be processed per day. For simplicity, the number of samples that can be processed per day was fixed at 100. This assumption implies that the cost per sample will not increase or decrease when fewer or more samples per day are screened. It was estimated that the cost of materials for 50 individuals or pooled samples equaled US$ 4. For more details on the itemized cost assessment see S1 Table. The daily salary equaled US$ 13.7 for each team member. When samples were individually processed, a team consisted of three laboratory technicians and one nurse. When a pooled examination strategy was applied, we assumed two technicians were required rather than three. Data entry clerks were paid US $1.1 per 100 data records entered. The daily cost of car rental (including driver) was US$ 66.0 and the cost for fuel (20 L) was estimated at US$ 15.8. Two schoolteachers were paid US$ 7.2 each to support the survey team in informing the students about the survey, facilitating the selection of students, and sample collection.

We determined the number of days required for each of the four activities (work at school, administration, travel, and days off) within a period of 12 weeks (84 days) for three scenarios of school accessibility. S2 Fig illustrates the activities over a 12-week period when school accessibility was low. In this scenario, there are 45 days of work at school, 21 days off, and 9 days for either travel or administration. S3 and S4 Figs illustrate the activities over a 12-week period when school accessibility is moderate and high, respectively. In these scenarios, the total number of five schools per district remained unchanged, and hence there are days that fewer schools per day are visited. For example, in the scenario of moderate school accessibility, two schools per day will be surveyed in the first two days, followed by one day where only one school is visited. In that case, the daily cost for a poorly accessible school was used. Finally, the number of days and the cost per days were then multiplied for each activity separately, to obtain the total operational cost.

We performed a one-way sensitivity analysis in which the cost for materials, salary, fee for school teachers, data entry, rent of car and fuel transport was increased and decreased by 10%. This univariate sensitivity analysis was applied for both individual and pooled examination strategies and the three scenarios of school accessibility, separately.

## Results

### Prevalence and intensity of STH and *S*. *mansoni* infections

Eggs of STH or *S*. *mansoni* were found in 354 out of the 2,650 (13.4%) subjects. The predominant helminth species was hookworm (6.2%, n = 113), followed by *A*. *lumbricoides* (4.3%, n = 164) and *S*. *mansoni* (2.6%, n = 70). The least prevalent was *T*. *trichiura* (0.8%, n = 22). The overall mean FEC was 45.1 EPG for *A*. *lumbricoides*, 17.5 EPG for hookworm, 1.6 EPG for *S*. *mansoni* and 1.8 EPG for *T*. *trichiura*. At the level of the schools, at least one parasite was found in 41 out of 53 (77.4%) schools, and as illustrated in S2 Table, both prevalence and intensity of infections ranged widely across the schools (*T*. *trichiura*: 2.0–26.0%, 0.5–55.2 EPG; *S*. *mansoni*: 2.0–36.0%, 0.5–35.5 EPG; *A*. *lumbricoides*: 2.0–50.0%, 0.5–1,168.8 EPG; and *hookworms*: 2.0–78.0%, 0.5–535.2 EPG).

### Qualitative and quantitative assessment of STH and *S*. *mansoni* infections

Tables 1 and 2 summarize the sensitivity at the level of the pools and the schools, respectively. The sensitivity was set at 100% when at least one parasite egg was detected in a sample or school for both strategies and the sensitivity of both strategies were compared against this target. Generally, the examination of pooled samples resulted in a significantly lower sensitivity for each of the four helminths. At the level of the pools, the sensitivity for a pooled examination strategy ranged from 16.7% for *T*. *trichiura* to 61.8% for hookworms, whereas for an individual examination strategy the sensitivity was at least 90% for all helminths. When determined at the school level, the sensitivity remained roughly unchanged, for a pooled examination strategy it ranged from 22.2% for *T*. *trichiura* to 75.0% for *S*. *mansoni* and was significantly lower than the sensitivity of individual examination strategy (sensitivity >89%).

**Table 1:** The sensitivity of individual and pooled examination strategies at the level of the pools. A pool of 10 individual samples is defined positive if either at least one egg is found following a pooled examination strategy or the mean fecal egg counts of the corresponding individual samples is non-zero.

Parasite species	Number of positive pools	Sensitivity (%) [95% CI]		p–value for pair-wise comparison
		Individual samples	Pooled samples	
***A*. *lumbricoides***	80	90.0	55.0	<0.001
		[83.8–96.2]	[43.8–66.2]	
***T*. *trichiura***	12	91.7	16.7	<0.001
		[75.0–100]	[0–41.7]	
**Hookworms**	68	92.6	61.8	<0.001
		[85.3–98.5]	[50.0–73.5]	
***S*. *mansoni***	29	100	51.7	<0.001
		[100–100]	[34.5–69.0]	

**Table 2:** The sensitivity of individual and pooled examination strategies at the level of the schools. A school is defined positive if either at least one egg is found in any of the individual samples or any of the 5 pooled samples.

Parasite species	Number of positive schools	Sensitivity (%) [95% CI]		p–value for pair-wise comparison
		Individual samples	Pooled samples	
***A*. *lumbricoides***	32	93.8	62.5	<0.001
		[84.4–100]	[46.9–78.1]	
***T*. *trichiura***	9	88.9	22.2	0.005
		[66.7–100]	[0–55.6]	
**Hookworms**	21	100	61.9	<0.001
		[100–100]	[42.9–81.0]	
***S*. *mansoni***	8	100	75.0	<0.001
		[100–100]	[37.5–100]	

**Fig 1:**
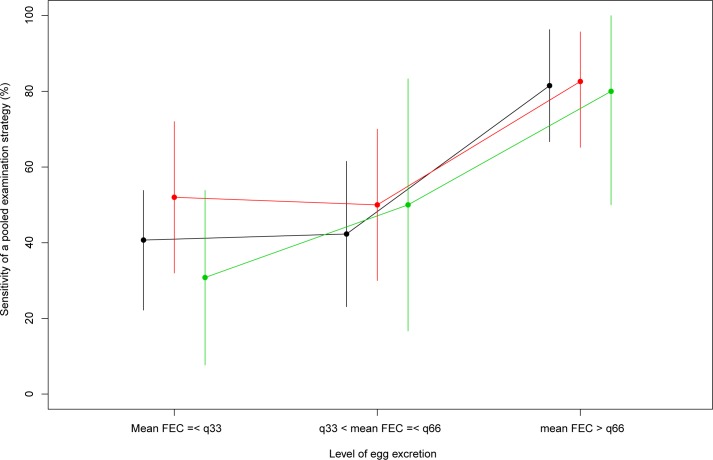
The sensitivity of a pooled examination strategy for *Schistosoma mansoni*, *Ascaris lumbricoides*, and *hookworms*. The green line/dots represent *S*. *mansoni*, the black line/dots represent *A*. *lumbricoides*, and the red line/dots represent hookworms infections. The dots represent the point estimates, whereas the vertical lines represent the 95% confidence interval. The values for the 33^rd^ (q33) and the 66^th^ quantile (q66) were 7.2 eggs per gram of stool (EPG) and 15.6 EPG for *S*. *mansoni*, 7.4 EPG and 34.3 EPG for *A*. *lumbricoides*, 4.8 EPG and 26.9 EPG for hookworm. Given the low number of cases, *Trichuris* was not included in the graph.

As illustrated in Fig 1, the probability of detecting helminth eggs increased with higher levels of egg excretion for *A*. *lumbricoides* (level 1: 40.7% vs. level 2: 42.3% vs. level 3: 81.5%), hookworms (level 1: 52.0% *vs*. level 2: 50.0% *vs*. level 3: 82.6%) and *S*. *mansoni* (level 1: 30.8% *vs*. level 2: 50.0% *vs*. level 3: 80.0%). The pair-wise comparison revealed only a significant difference between levels 1 and 3 (*p* = 0.006), and levels 2 and 3 (*p* = 0.012) for *A*. *lumbricoides*. Since there were only 12 cases of *T*. *trichiura*, variation in sensitivity across the levels of egg excretion was not assessed.

**Fig 2:**
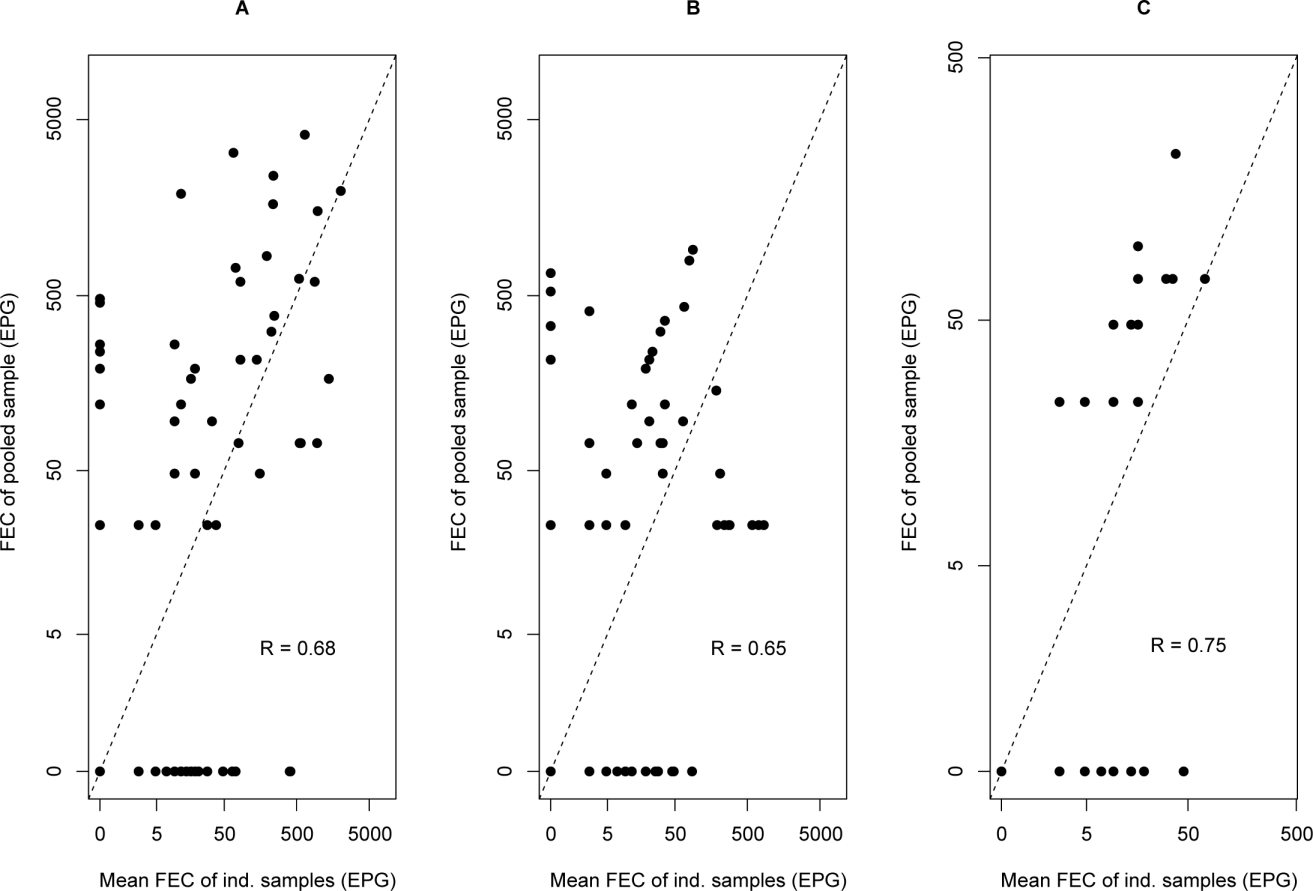
Agreement in fecal egg counts of helminth infections between individual and pooled samples. The scatter plots represent the agreement in mean individual fecal egg counts (FEC; expressed in eggs per gram of stool (EPG)) and pooled FEC for *Ascaris lumbricoides* (Panel A), hookworms (Panel B), and *Schistosoma mansoni* (Panel C). Each dot represents the FEC in a pooled sample (y-axis) and the mean FECs of the corresponding individual FECs (x-axis). The degree of correlation for each plot is based on Pearson’s correlation coefficient (R). For each of the three helminth species, a significant positive correlation was observed (*p* <0.001). Given the low number of cases, *Trichuris* was not included in the graph.

Overall, there was a significant positive correlation between the mean FECs of individual samples and the FECs of the pooled samples for *A. lumbricoides* (0.68, p <0.001), hookworm (0.65, p <0.001), and *S. mansoni* (0.75, p <0.001) (Fig 2). Given the low number of cases at the pooled level (n = 12), the correlation in FECs between examination strategies was not determined for *T. trichiura*.

The mean FEC based on a pooled examination strategy were generally higher than those based on an individual strategy (Table 3). A significant difference between FECs derived from individual and pooled examination strategies was observed for *A*. *lumbricoides*, (FEC_individual_ = 45.1 EPG *vs*. FEC_pooled_ = 93.9 EPG, *p* = 0.03) and *S*. *mansoni* (FEC_individual_ = 1.6 EPG *vs*. FEC_pooled_ = 3.4 EPG, *p* = 0.02).

**Table 3:** Intensity of four helminth species infections based on individual and pooled examination strategies.

Parasite species	N	Mean FEC at the pool level [95% CI]		Difference [95% CI]	P-value of pair-wise comparison
		Individual samples	Pooled samples		
***A*. *lumbricoides***	265	45.1	93.9	-48.8	0.03
		[24.0; 71.3]	[49.8; 151.8]	[-97.2; - 9.00]	
***T*. *trichiura***	265	1.8	2.1	-0.3	0.95
		[0.2; 4.1]	[0.0; 6.2]	[-4.0; 2.6]	
**Hookworms**	265	17.5	28.5	-11	0.18
		[8.8; 28.3]	[16.6; 42.8]	[-27.2; 4.7]	
**S. *mansoni***	265	1.6	3.4	-1.8	0.02
		[0.9; 2.6]	[1.5; 5.8]	[-3.5; -0.2]	

### Time required for sample testing

The total time to prepare and read 2,450 stool samples individually equaled 198h 16min, compared to 53h 50min when samples were processed in pools; a reduction of 72.8%. Considerable inter-team variation was observed in time required to prepare and screen samples (Table 4). The mean time for the preparation of ten individual Kato-Katz thick smears and pools of ten individual stool samples ranged from 10.3 min to 31.7 min, and from 2.4 min to 8.8 min, respectively. The reading of the Kato-Katz thick smears ranged from 2.2 min to 4.4 min for the examination of individuals, and 2.7 min to 8.2 min for the examination of pools. Across the teams the reduction in time when using a pooled strategy ranged from 50.1% to 82.0%.

**Table 4:** The estimated time for a both an individual and a pooled examination strategy. Time is expressed in minutes and with the corresponding standard deviation (SD) reported.

ID team	Individual examination				Pooled examination					Reduction in time when a pooled examination strategy is applied (%)^d^
	Number of individual samples	Mean (SD) time to prepare 10 KKs (min)	Mean (SD) time to read a KK of an individual (min)	Total time to prepare and read individual samples^a^	Number of pooled samples	Mean (SD) time to prepare a pool of 10 individual samples	Mean (SD) time to prepare 10 KKs (min)^b^	Mean (SD) time to read a KK of a pooled sample (min)	Total time to prepare and read pooled samples^c^	
**11**	350	13 (1.2)	3.7 (0.7)	29 h 6 min	35	2.4 (0.8)	13 (1.2)	5.3 (1.3)	5 h 15 min	82.0
**12**	550	31.7 (3.3)	4.4 (0.8)	69 h 26 min	55	8.8 (1.2)	31.7 (3.3)	5.2 (0.9)	15 h 43 min	77.4
**13**	800	10.3 (5.3)	2.4 (0.7)	45 h 39	80	7.8 (2.4)	10.3 (5.3)	8.2 (2.0)	22 h 46 min	50.1
**14**	300	13.3 (2.4)	2.2 (0.9)	17 h 35 min	30	2.6 (0.9)	13.3 (2.4)	2.7 (0.7)	3 h 17 min	81.3
**15**	450	11.9 (2.8)	3.7 (0.9)	36 h 30 min	45	4.1 (3.1)	11.9 (2.8)	3.8 (1.1)	6 h 49 min	8 81.3
				**198 h 16 min**					**53 h 50 min**	**72.8**

KK: single Kato-Katz thick smear; a: Total time to prepare and read individual samples = [(number of individual samples x mean time to prepare 10 KK)/10 + number of individual samples x mean time to read a KK of an individual sample)] / 60; b: As the time to prepare 10 KKs is not affected by the examination strategy, the mean time to prepare 10 KK was the same for both an individual and a pooled examination strategy; c: Total time to prepare and read pooled samples = [(number of pooled samples x mean time to prepare 10 KK)/10 + number of pooled samples x mean time to read a KK of a pooled sample)] / 60; d: Reduction in time when a pooled examination strategy is applied = 100% x (1—total time to prepare and read pooled samples / total time to prepare and read individual samples)

### Estimation of operational costs

The cost per day of the four activities when samples were individually examined and when schools are poorly accessible are summarized in Table 5. The estimated daily costs were US$ 155.6 for work at school, US$ 136.6 for a day of administration and a day of travel, and US$ 128.7 for a day-off. The differences in daily cost per activity can be explained by differences in usage of material (only required for work at school), payment of fees to school teachers (only applicable for work at school), and the amount of fuel (less fuel required on days off). The cost breakdown for one day of work at school across the three levels of school accessibility when samples are individually examined is summarized in Table 6. The daily cost increases from US 155.6 for poorly accessible schools to US$ 190.3 for moderately accessible schools to US$ 225.1 for highly accessible schools. Table 5 reports the cost breakdown for the four activities at a poorly accessible school using a pooled strategy. Table 6 reports the costs of work comparing the different levels of accessibility under a pooled strategy. These two tables show that costs are lower compared to an individual examination strategy, and these differences are due to lower usage of materials and less data entry (one tenth of an individual examination strategy). Moreover, since the workload to process samples can be covered by only 2 laboratory technicians, salary costs were reduced. The number of days of work at school, administration, travel and days-off over an 84-day period are summarized in S2 Fig (poor accessibility of schools), 3 (moderate accessibility of schools) and 4 (high accessibility of schools). When schools are poorly accessible, a team will spend 45 days (53.6%) working in schools and 9 days for administration (10.3%) and travel (10.3%), and will have 21 days-off (25.0%). When the schools are moderately and highly accessible the number of days of working in schools equaled 36 (42.9%) and 32 (38%), respectively. The number of days dedicated to administration and travel equaled 12 (14.3%; moderate school accessibility) and 16 days (19%, high school accessibility), the number of days-off equaled 24 (28.6%; moderate school accessibility) and 20 (23.8%; high school accessibility).

**Table 5:** The breakdown of the daily costs for four activities when the school accessibility is poor. In this scenario of school accessibility, only one school can be screened per day. Fifty subjects were randomly selected per school.

Cost	Cost per unit (US$)	Activity							
		Work at school		Administration		Travel		Day off	
		N of units	Cost (US$)	N of units	Cost (US$)	N of units	Cost (US$)	N of units	Cost (US$)
**Individual examination strategy**									
Material	0.08	50	4.0	0	0.0	0	0.0	0	0.0
Salary									
*Team member*	13.7	4	54.8	4	54.8	4	54.8	4	54.8
*Local clerk*	1.1	0.5	0.6	0	0.0	0	0.0	0	0.0
Fee school teacher	7.2	2	14.4	0	0.0	0	0.0	0	0.0
Rental vehicle	66	1	66.0	1	66.0	1	66.0	1	66.0
Fuel (20 L)	15.8	1	15.8	1	15.8	1	15.8	0.5	7.9
**Total**			**155.6**		**136.6**		**136.6**		**128.7**
**Pooled examination strategy**									
Material	0.08	5	0.4	0	0.0	0	0.0	0	0.0
Salary									
*Team member*	13.7	3	41.1	3	41.1	3	41.1	3	41.1
*Local clerk*	1.1	0.05	0.1	0	0.0	0	0.0	0	0.0
Fee school teacher	7.2	2	14.4	0	0.0	0	0.0	0	0.0
Rental vehicle	66	1	66.0	1	66.0	1	66.0	1	66.0
Fuel (20 L)	15.8	1	15.8	1	15.8	1	15.8	0.5	7.9
**Total**			**137.8**		**122.9**		**122.9**		**115.0**

**Table 6:** The cost for one day of work at school across three levels of school accessibility. The accessibility of the schools was defined as poor, moderate, and high. Schools were poorly accessible when only one school per day can be surveyed. Schools were moderately and highly accessible when 2 and 3 schools per day can be surveyed, respectively. In each scenario of school accessibility, 50 subjects are randomly selected per school.

Cost	Cost per unit (US$)	Accessibility of school					
		Poor		Moderate		High	
		N of units	Cost (US$)	N of units	Cost (US$)	N of units	Cost (US$)
**Individual examination strategy**							
Material	0.08	50	4.0	100	8.0	150	12.0
Salary							0.0
*Team member*	13.7	4	54.8	4	54.8	4	54.8
*Local clerk*	1.1	0.5	0.6	1	1.1	1.5	1.7
Fee school teacher	7.2	2	14.4	4	28.8	6	43.2
Rental vehicle	66	1	66.0	1	66.0	1	66.0
Fuel (20 L)	15.8	1	15.8	2	31.6	3	47.4
**Total**			**155.6**		**190.3**		**225.1**
**Pooled examination strategy**							
Material	0.08	5	0.4	10	0.8	15	1.2
Salary							0.0
*Team member*	13.7	3	41.1	3	41.1	3	41.1
*Local clerk*	1.1	0.05	0.1	0.1	0.1	0.15	0.2
Fee school teacher	7.2	2	14.4	4	28.8	6	43.2
Rental vehicle	66	1	66.0	1	66.0	1	66.0
Fuel (20 L)	15.8	1	15.8	2	31.6	3	47.4
**Total**			**137.8**		**168.4**		**199.1**

The total operational costs for one team to be on the road for 12 weeks across the different levels of school accessibility are summarized in Table 7. When samples are processed individually, the estimated operational costs were US$ 12,161.3 when schools are poorly accessible, US$ 12,801.0 when schools are moderately accessible and US$ 13,590.8 when schools are highly accessible. Applying a pooled examination strategy reduced the costs by approximately 11%, regardless of the accessibility of schools.

**Table 7:** The total operational cost for assessing stool samples across three levels of school accessibility. The accessibility of the schools was defined as poor, moderate and high. Schools are poorly accessible when only one school per day can be surveyed. Schools are moderately and highly accessible when 2 and 3 schools per day can be surveyed, respectively. In each scenario of school accessibility, 50 subjects are randomly selected per school.

	N of days	Individual samples		Pooled samples		Reduction in costs (%)
		Cost per day (US$)	Cost (US$)	Cost per day (US$)	Cost (US$)	
**Poor school accessibility**						
Work at school	45	155.6	6,999.8	137.8	6,199.0	
Administration	9	136.6	1,229.4	122.9	1,106.1	
Travel	9	136.6	1,229.4	122.9	1,106.1	
Day-off	21	128.7	2,702.7	115.0	2,415.0	
	84		12,161.3		10,826.2	11.0
**Moderate school accessibility**						
Work at school						
1 school	12	155.6	1,866.6	137.8	1,653.1	
2 schools	24	190.3	4,567.2	168.4	4,041.8	
Administration	12	136.6	1,639.2	122.9	1,474.8	
Travel	12	136.6	1,639.2	122.9	1,474.8	
Day off	24	128.7	3,088.8	115.0	2,760.0	
	84		12,801.0		11,404.5	10.9
**High school accessibility**						
Work at school						
2 schools	16	190.3	3,044.8	168.4	2,694.6	
3 schools	16	225.1	3,600.8	199.1	3,185.0	
Administration	16	136.6	2,185.6	122.9	1,966.4	
Travel	16	136.6	2,185.6	122.9	1,966.4	
Day off	20	128.7	2,574.0	115.0	2,300.0	
	84	128.7	13,590.8		12,112.4	10.9

**Fig 3:**
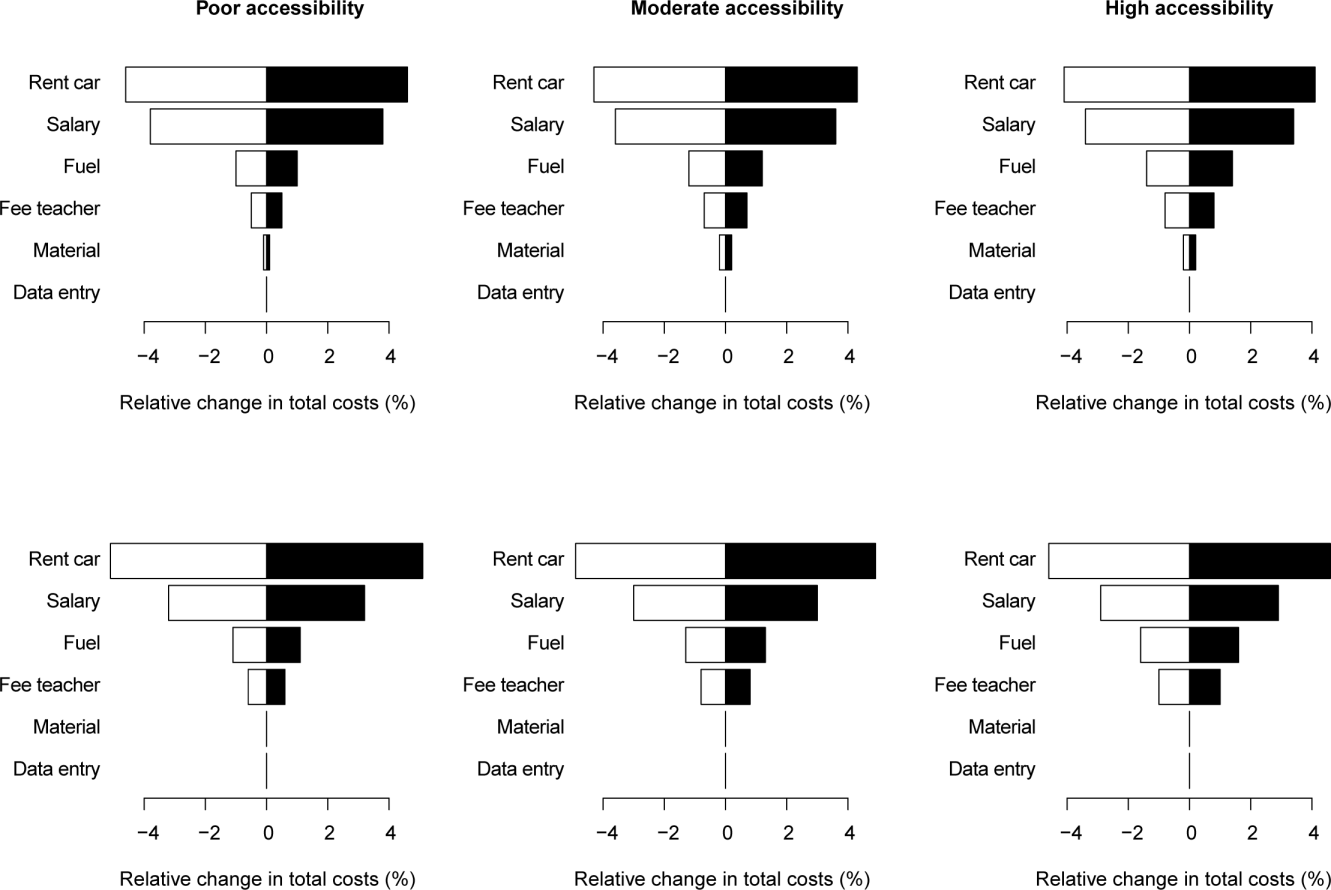
The impact of six expense types on the total operational costs. Each of the six tornado plots represents the impact of material, salary, data entry, school teachers’ fees, car rental, and fuel on the total operational costs for both an individual (top graphs) and a pooled (bottom graphs) examination strategy for three levels of school accessibility (poor, moderate, and high). White bars represent the relative change in operational costs when the expenses are increased by 10%, black bars represent the relative change in operational costs when the expenses are decreased by 10%. The relative change in operational costs equaled 100% x (1 –operational costs when the expenses varied with 10% / initial operational costs). A negative value indicates an increase in operational costs, a positive indicates a decrease in operational costs.

**Fig 4:**
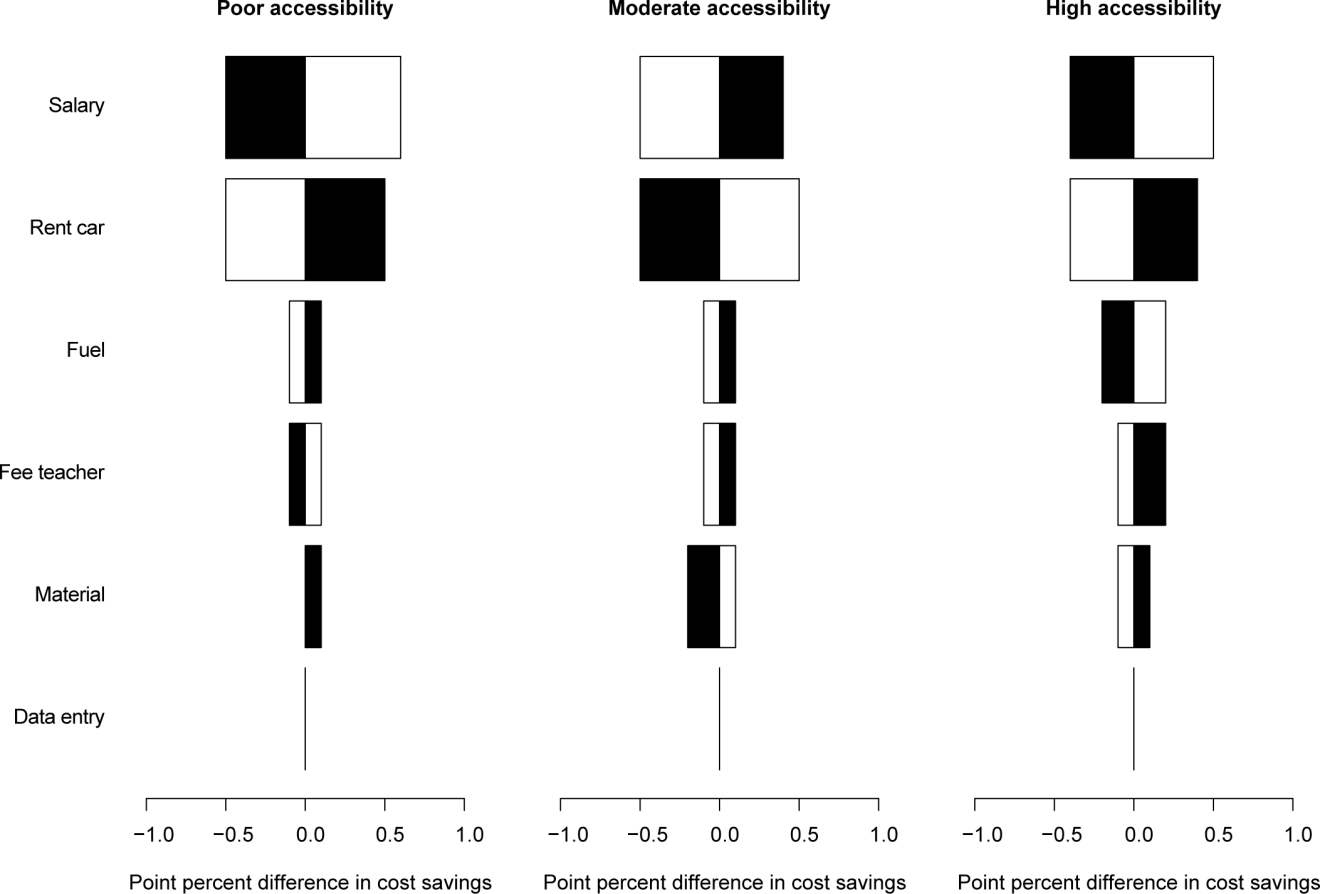
The impact of the six expense types on cost-saving when a pooled examination strategy was applied. Each of the 3 tornado plots represents the impact of material, salary, data entry, school teachers’ fees, car rental, and fuel on the cost-saving when a pooled examination strategy was applied for three levels of school accessibility (poor, moderate, and high). White bars represent the relative change in cost-saving when the expenses were reduced by 10%, black bars represent the relative change in cost-saving when the expenses were increased by 10%. A negative value indicates that the cost-saving increased, a positive value indicates a decrease in cost-saving.

The one-way sensitivity analysis, presented in the Figs 3 and 4, demonstrates the main cost drivers. Overall, car hire had the largest impact on total costs, followed by salaries (Fig 3). Varying these parameters resulted in a relative change in operational costs of approximately 4% and 3%, respectively. The impact of the other parameters did not exceed 2% (fuel: 0.7%– 1.6%; material: 0.0–0.2%; data entry: 0.0–0.0%).

The impact of car rental costs was consistent across examination strategies and levels of school accessibility. The impact of salary variance was similar for the three levels of accessibility, but was slightly less pronounced for an individual examination strategy (~3.0%) compared to a pooled examination strategy (~3.6%). Despite these differences in total operational costs, both parameters had little impact on the cost-savings effect of a pooled examination strategy (less than 1% reduction; (Fig 4)).

## Discussion

SCH and STH programs rely heavily on large-scale surveys to initiate and monitor their success [22]. Initial treatment frequency is determined by the prevalence of infection at baseline, prior to treatment, based on WHO guidelines [1]. Ongoing monitoring and evaluation primarily uses intensity of infection as an indicator for the program’s progress. Under either the individual or pooled approach, large-scale surveys constitute an important cost for governments and funders, which in resource-limited countries present major challenges. Various studies have provided insights on minimizing the operational costs for individual diagnosis of helminths, while ensuring accurate results and subsequently, correct programmatic decisions [23]. To date, the diagnostic strategy of pooling stool to reduce costs has not been fully explored for STH and SCH in humans [15–18], as studies were based on a small number of samples collected in confined geographical areas where transmission was moderate to high. Therefore, our group tested the applicability of a pooling strategy under field conditions during the national mapping of STH and SCH in Ethiopia. We compared individual and pooled examination strategies for the detection and quantification of STH and intestinal schistosomiasis (caused by *S*. *mansoni*) at a scale that is unprecedented in the literature and in area were transmission was low. Finally, we compared the time for sample testing, and the total operational costs for both strategies.

Overall, our findings on the diagnostic performance are in line with previous small-scale laboratory studies [15–18], confirming that a pooled strategy provides comparable estimates of population infection intensity, but that it often fails to detect infections, particularly those that are light. At this stage, it remains premature to make any formal recommendations on a pooled approach in a programmatic setting. We evaluated a pooling approach during an early phase of a STH and SCH program (mapping of disease) in a low transmission area applying only one diagnostic method (a single Kato-Katz thick smear) and one pool size (10 individual samples). It has been shown that the sensitivity of a pooled examination strategy is a function of the number of individual samples pooled (sensitivity inversely correlated with the number of individual samples) and the intrinsic sensitivity of the diagnostic technique [17,18]. As a consequence of this, pooling 10 individual samples and testing with a single Kato-Katz thick smear, a technique with poor sensitivity [24], may not be ideal to assess the intensity and prevalence of infections in all possible scenarios of STH and SCH epidemiology and phases of the program. Complementary studies evaluating pooling of samples in varying scenarios of endemicity, program phase, and diagnostic effort (number of samples pooled and analytic sensitivity) are welcomed to inform program managers on when and how to best pool samples. Given that it would be impossible to field test each of these scenarios, one could complement field studies with *in silico* approaches. Such an approach are best illustrated by the recent study by Lo et al. (e.g., reference 18). In this study, field data were used to inform a micro-simulation study. This *in silico* study was designed to verify whether pooling held promise for drawing programmatic conclusions across scenarios of endemicity other than those observed in the field. For application of a pooled approach in assessing the prevalence of infections, it is also necessary to develop and validate statistical approaches that allow the estimation of the true underlying prevalence based on the results of a pooled examination strategy. A variety of methods have been described for this, and they differ based on how the inference is drawn (frequentist vs. Bayesian approach), assumptions on the diagnostic performance (perfect vs. imperfect diagnostic techniques), number of samples pooled (fixed number *vs*. variable number) and input data (binary inputs vs. counts) With a few exceptions, these methodologies were initially developed for diseases other than STH or SCH [25–28].

Our results on the time required for testing demonstrated that a pooling strategy reduced the time to prepare and read slides under field settings by 72.8%. A previous study which pooled five individual samples reported a similar reduction in time (~70%; Kure et al., 2015), and this highlights that the reduction in laboratory time is likely not a linear function of the number of samples pooled. Between field teams there was a large variation in reduction in laboratory time between the examination strategies, ranging from 50.1% to 82.0%. This variation can by explained by a series of factors, such as the experience of technicians, school set-up, and varying issues related to the working environment. In general, a large proportion of the time for sample testing is dedicated to reading slides: reading a slide takes more than half of the total time to test an individual stool sample (Table 4). Efforts to develop and validate easy-to-use and point-of-care technology that allows electronic imaging of slides, and subsequently automated egg counting should be further encouraged [29].

Our results indicate that, despite a reduction in sample testing time of ~73%, the pooling strategy has relatively little impact on total survey costs (total operational costs were reduced by ~11%). The cost of any survey likely depends on diagnostic technique/s used and survey design [24,30–32]. The one-way sensitivity analysis on the different sources of costs revealed little to no variation in the relative cost-savings when a pooled examination strategy was used. Rather, our results indicated that the total operational costs were mainly impacted by logistical factors such as obtaining permission from the district offices and being constrained to the days children are at school. These factors incur additional costs for vehicle rental and survey team salaries, which affected the total operational cost for both strategies. In this regard, our observations indicated that under the different scenarios of school accessibility the teams spend between 36% and 44% of the total days off work when one and three schools are sampled per day. As recently highlighted by Turner and colleagues [33] the cost of a Kato-Katz thick smear varies considerably due to factors such as the method of collection (processing samples on site vs. examining samples next day off-site), the number of sites sampled per day (increases cost), the number of samples collected per site (decreases cost), variation in personnel, and adjustment of microscope costs (microscope used for other activities vs. microscope exclusively used for the STH/SCH survey). Given the number of schools per woreda (n = 5), subjects per school (n = 50), the operational steps in the field (S1 and S2 Figs), their corresponding costs (Table 7) and a survey period of 12 weeks, the estimated cost per single Kato-Katz thick smear on an individual stool sample varies from US$ 3.4 when 3 schools are surveyed per day (total number of school children = 4,000) to US$ 5.4 when one school is surveyed per day (total number of children = 2,250). Under the same scenario, the cost for a single Kato-Katz thick smear when a pooled examination strategy is applied increases approximately tenfold (US$ 48.1 when one school is surveyed per day: US$ 30.2 when 3 schools are surveyed per day), indicating that the way samples are examined (individual vs. pooled) should also be considered when costs of the Kato-Katz thick smear are estimated. These differences are explained by the low number of Kato-Katz thick smears (1x a single Kato-Katz thick smear is processed from one pooled sample *vs*. 10x a single Kato-Katz thick smears from 10 individual samples) and the relatively low reduction in total operational cost when samples are pooled. Finally, the total operational costs were estimated for this specific survey in an Ethiopian setting, and care should be taken when extrapolating to any other national programs. Consequently, it is necessary to compare operational costs of both strategies across a variety of scenarios of national program management to determine whether and when pooling is worthwhile considering.

In conclusion, we identified that a pooled strategy provided comparable results for infection intensity, but that it lacks sensitivity and therefore may perform poorly at estimating infection prevalence. A pooled examination strategy resulted in a reduction of 73% of time spent for sample testing, but this only resulted in a reduction of 11% in total operational costs. Based on these findings we conclude that a pooled examination strategy holds some promise for the rapid assessment of intensity of STHs and schistosome infections in a programmatic setting, but that does not result in a major cost-saving opportunity. To make any formal recommendations on a pooled approach, further investigation is required to determine when and how pooling can be utilized. For prevalence-based assessments, such work should also include validation of statistical methods to estimate prevalence based on a pooled examination strategy. Finally, operational costs should be compared different scenarios of national program management.

## Supporting information

S1 Fig Procedure to obtain pools of 10 individual stool samples. Fifty individual samples were arranged in five rows of ten individual samples. From each individual of the same row, 1g of stool was transferred into a new pre-labeled stool cup, and the pool was thoroughly mixed with a wooden spatula until the color of the mixture became uniform.
(TIF)

S2 Fig The days for administration, work at school, travel and days-off when schools were poorly accessible. (TIF)

S3 Fig The days for administration, work at school travel and days-off when schools are moderately accessible. (TIF)

S4 Fig The days for administration, work at school, travel and days-off when schools are highly accessible. (TIF)

S1 Table The itemized cost assessment for the examination of 1,000 individual stool samples. (DOCX)

S2 Table The prevalence and intensity of helminth infections in 53 schools of the Amhara Regional State. (DOCX)

S3 Table The absolute and proportional changes in total operational costs when the costs varied by 10%. (DOCX)
