# Supplementary figures and images for: Comparison of individual and pooled diagnostic examination strategies during the national mapping of soil-transmitted helminths and Schistosoma mansoni in Ethiopia
Source: PLoS Negl Trop Dis. 2018 Sep 10;12(9):e0006723. doi: 10.1371/journal.pntd.0006723 (PMC6147605; doi:10.1371/journal.pntd.0006723)

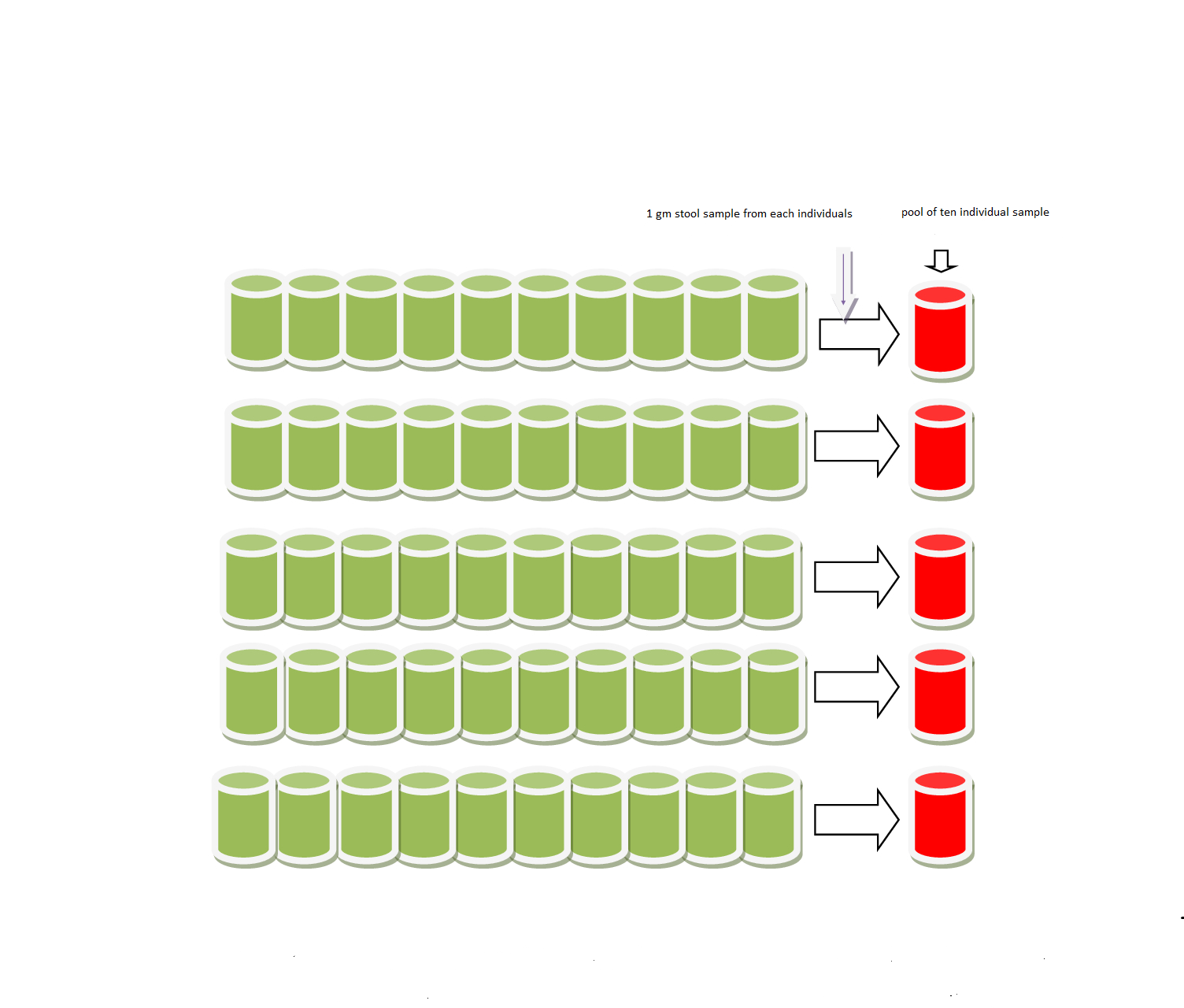

Supplement: S1 Fig — Fifty individual samples were arranged in five rows of ten individual samples. From each individual of the same row, 1g of stool was transferred into a new pre-labeled stool cup, and the pool was thoroughly mixed with a wooden spatula until the color of the mixture became uniform. (TIF) [file pntd.0006723.s001.tif]

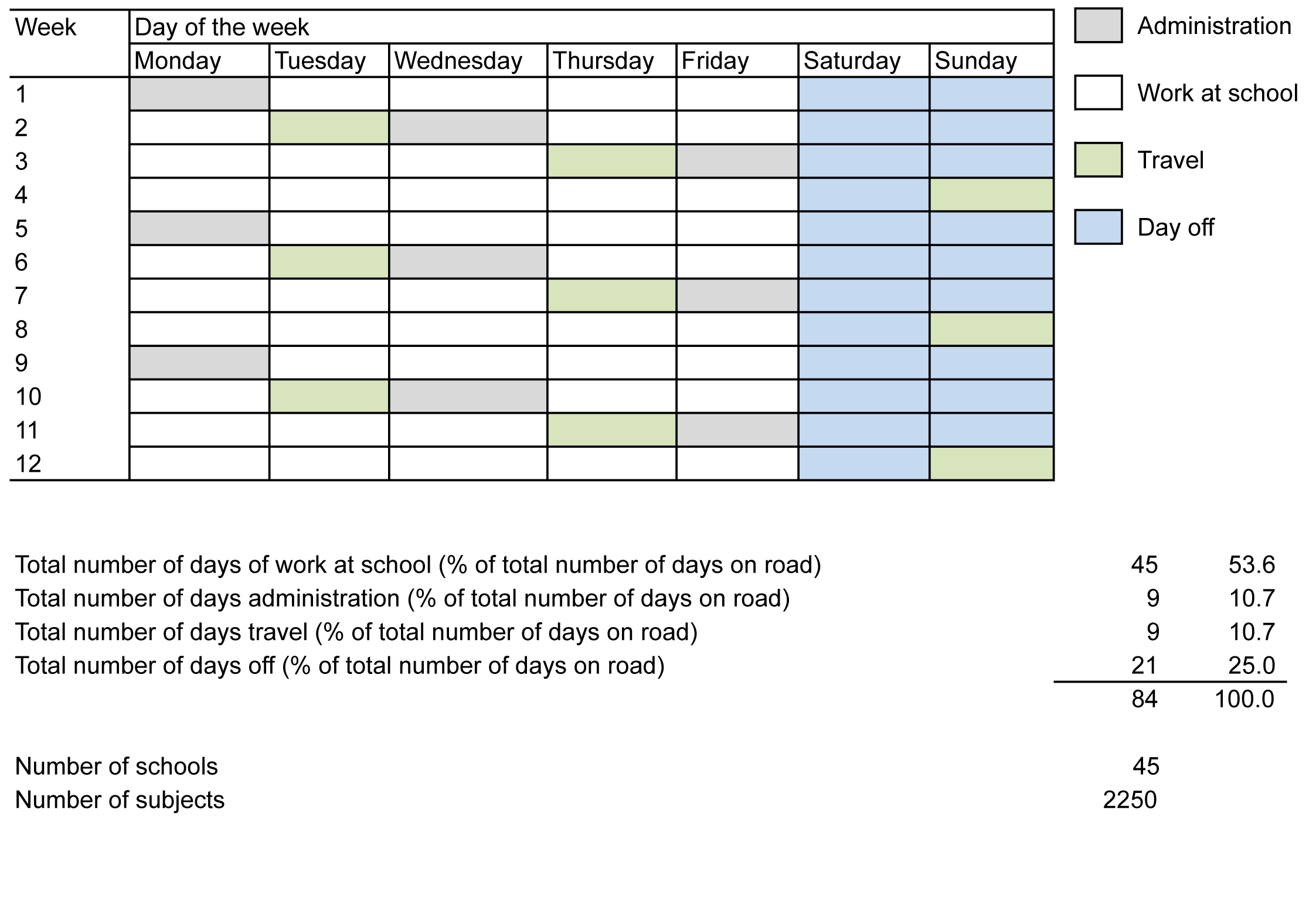

Supplement: S2 Fig — (TIF) [file pntd.0006723.s002.tif]

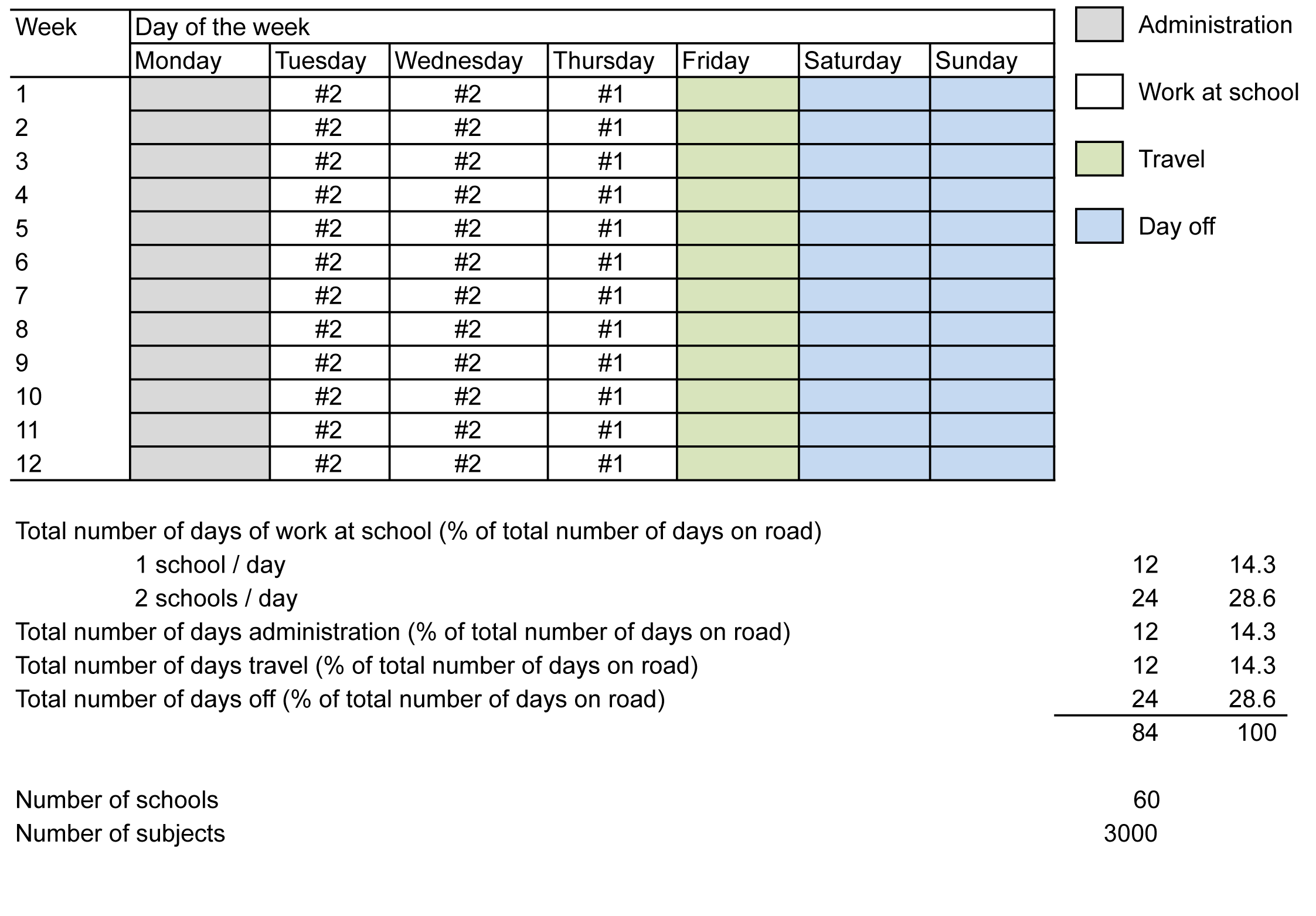

Supplement: S3 Fig — (TIF) [file pntd.0006723.s003.tif]

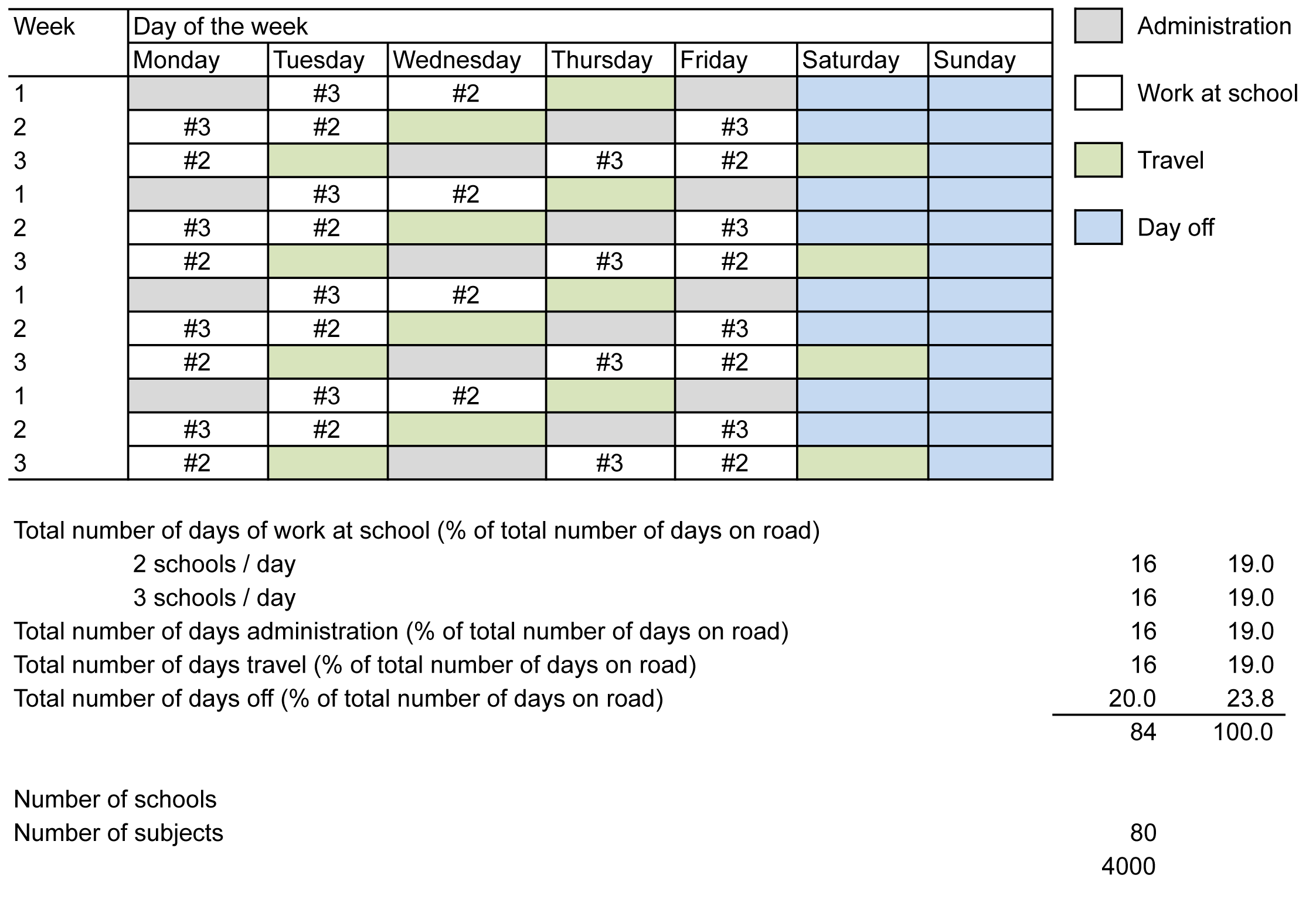

Supplement: S4 Fig — (TIF) [file pntd.0006723.s004.tif]
