# Supplementary material for: Comparison of individual and pooled diagnostic examination strategies during the national mapping of soil-transmitted helminths and Schistosoma mansoni in Ethiopia
Source: PLoS Negl Trop Dis. 2018 Sep 10;12(9):e0006723. doi: 10.1371/journal.pntd.0006723 (PMC6147605; doi:10.1371/journal.pntd.0006723)
Supplement: S1 Table — (DOCX) [file pntd.0006723.s005.docx]

**Supplementary Table 1. The itemized cost assessment for the examination of 1,000 individual stool samples**

|  | **Item** | **Cost per unit in birr** | **Cost per unit in US$** | **Number of days / year used** | **Life expectancy in years** | **Number of individual samples processed per day** | **Cost per 1,000 individual samples in US$** |
| --- | --- | --- | --- | --- | --- | --- | --- |
| **Equipment** | | | | | | |  |
|  | Microscope | 62,000 | 2,863.74 | 100 | 5 | 100 | 57.27 |
|  | Slide box | 100 | 4.62 | 100 | 2 | 100 | 0.23 |
|  | Forceps | 50 | 2.31 | 100 | 2 | 100 | 0.12 |
|  | Petri dish | 60 | 2.77 | 100 | 2 | 100 | 0.14 |
|  | Buckets | 40 | 1.85 | 100 | 2 | 100 | 0.09 |
|  | Urine filtration kit | 300 | 13.86 | 100 | 2 | 100 | 0.69 |
|  | Scissors | 25 | 1.15 | 100 | 2 | 100 | 0.06 |
|  | Beaker of 1000 ml | 60 | 2.77 | 100 | 2 | 100 | 0.14 |
|  | Smartphone | 5,000 | 230.95 | 100 | 4 | 100 | 5.77 |
|  | Ice box | 600 | 27.71 | 100 | 2 | 100 | 1.39 |
|  | Bottle washer | 40 | 1.85 | 100 | 2 | 100 | 0.09 |
|  | Jeri can of 25 liter | 65 | 3.00 | 100 | 2 | 100 | 0.15 |
|  | Torch | 160 | 7.39 | 100 | 2 | 100 | 0.37 |
|  | Tally counter | 250 | 11.55 | 100 | 10 | 100 | 0.12 |
|  | Puncher | 125 | 5.77 | 100 | 5 | 100 | 0.12 |
| **Supplies** | | | | | | |  |
|  | Laboratory book | 60 | 2.77 | 50 | 1 | 100 | 0.55 |
|  | Insecticide | 90 | 4.16 | 50 | 1 | 100 | 0.83 |
|  | Laboratory coat | 120 | 5.54 | 100 | 1 | 100 | 0.55 |
|  | Pen | 7 | 0.32 | 100 | 1 | 100 | 0.03 |
|  | Bench aid | 10 | 0.46 | 100 | 1 | 100 | 0.05 |
|  | Pencil | 1 | 0.03 | 100 | 1 | 100 | 0.00 |
|  | Pencil sharpener | 12 | 0.55 | 100 | 1 | 100 | 0.06 |
|  | Eraser | 5 | 0.23 | 100 | 1 | 100 | 0.02 |
|  | Umbro tap | 5 | 0.23 | 100 | 1 | 100 | 0.02 |
|  | Bio-safety bag | 5 | 0.23 | 100 | 1 | 100 | 0.02 |
|  | File folder | 45 | 2.08 | 100 | 1 | 100 | 0.21 |
|  | Applicator stick | 3 | 0.12 | 100 | 1 | 100 | 0.01 |
|  | Wooden spatula | 3 | 0.12 | 100 | 1 | 100 | 0.01 |
|  | Marker | 15 | 0.69 | 100 | 1 | 100 | 0.07 |
|  | t-shirt | 130 | 6.00 | 100 | 1 | 100 | 0.60 |
|  | Cap | 75 | 3.46 | 100 | 1 | 100 | 0.35 |
|  | Bag | 450 | 20.79 | 100 | 1 | 100 | 2.08 |
|  | Sleeping bag | 890 | 41.11 | 100 | 1 | 100 | 4.11 |
|  | Air freshener | 90 | 4.16 | 100 | 1 | 100 | 0.42 |
|  | Soap | 15 | 0.69 | 100 | 1 | 100 | 0.07 |
|  | Toilet paper | 10 | 0.46 | 100 | 1 | 100 | 0.05 |
|  | Labeler | 24 | 1.11 | 100 | 1 | 100 | 0.11 |
|  | Stamp pad | 12 | 0.55 | 100 | 1 | 100 | 0.06 |
|  | Note book | 12 | 0.55 | 100 | 1 | 100 | 0.06 |
|  | Plastic sheet | 4 | 0.18 | 100 | 1 | 100 | 0.02 |
|  | Powder soap | 15 | 0.69 | 100 | 1 | 100 | 0.07 |
|  | Cotton | 50 | 2.31 | 100 | 1 | 100 | 0.23 |
|  | Gloves | 75 | 3.46 | 100 | 1 | 100 | 0.35 |
|  | Tissue paper | 12 | 0.55 | 100 | 1 | 100 | 0.06 |
|  | News paper | 10 | 0.46 | 100 | 1 | 100 | 0.05 |
|  | Facemask | 5 | 0.23 | 100 | 1 | 100 | 0.02 |
|  | Waste disposal container | 5 | 0.23 | 100 | 1 | 100 | 0.02 |
|  | Microscopic slide | 1 | 0.06 | 100 | 1 | 100 | 0.01 |
|  | Urine container | 3 | 0.15 | 100 | 1 | 100 | 0.02 |
|  | Stool container | 3 | 0.15 | 100 | 1 | 100 | 0.02 |
|  | 10 cc syringe | 2 | 0.07 | 100 | 1 | 100 | 0.01 |
| **Reagents** | | | | | | |  |
|  | Urine dipstick | 50 | 2.31 | 100 | 1 | 100 | 0.23 |
|  | 70% alcohol | 70 | 3.23 | 100 | 1 | 100 | 0.32 |
|  | Malachite green | 250 | 11.55 | 100 | 1 | 100 | 1.15 |
|  | Bleach | 35 | 1.62 | 100 | 1 | 100 | 0.16 |
|  | **Total** |  |  |  |  |  | **79.81** |
