# Supplementary material for: Comparison of individual and pooled diagnostic examination strategies during the national mapping of soil-transmitted helminths and Schistosoma mansoni in Ethiopia
Source: PLoS Negl Trop Dis. 2018 Sep 10;12(9):e0006723. doi: 10.1371/journal.pntd.0006723 (PMC6147605; doi:10.1371/journal.pntd.0006723)
Supplement: S2 Table — (DOCX) [file pntd.0006723.s006.docx]

Supplementary Table 2. The prevalence and intensity of helminth infectionsin 53 schools of the Amhara Regional State.

| **ID School** | **N** | ***S. mansoni*** | |  | ***A. lumbricoides*** | |  | **Hookworms** | |  | ***T. trichiura*** | |
| --- | --- | --- | --- | --- | --- | --- | --- | --- | --- | --- | --- | --- |
|  |  | Prevalence (%) | Mean FEC (EPG) |  | Prevalence (%) | Mean FEC (EPG) |  | Prevalence (%) | Mean FEC (EPG) |  | Prevalence (%) | Mean FEC (EPG) |
| **1** | 50 | 0 | 0 |  | 0 | 0 |  | 18.0 | 11.0 |  | 0 | 0 |
| **2** | 50 | 0 | 0 |  | 0 | 0 |  | 24.0 | 13.4 |  | 0 | 0 |
| **3** | 50 | 0 | 0 |  | 2.0 | 1.9 |  | 42.0 | 98.4 |  | 0 | 0 |
| **4** | 50 | 0 | 0 |  | 12.0 | 277.9 |  | 6.0 | 5.8 |  | 0 | 0 |
| **5** | 50 | 12.0 | 3.8 |  | 0 | 0 |  | 18.0 | 42.7 |  | 26.0 | 55.2 |
| **6** | 50 | 0 | 0 |  | 0 | 0 |  | 24.0 | 59 |  | 0 | 0 |
| **7** | 50 | 0 | 0 |  | 2.0 | 4.3 |  | 78.0 | 535.2 |  | 2.0 | 1.9 |
| **8** | 50 | 0 | 0 |  | 12.0 | 200.2 |  | 0 | 0 |  | 2.0 | 29.8 |
| **9** | 50 | 0 | 0 |  | 0 | 0 |  | 40.0 | 43.7 |  | 0 | 0 |
| **10** | 50 | 0 | 0 |  | 8.0 | 4.8 |  | 2.0 | 1 |  | 0 | 0 |
| **11** | 50 | 0 | 0 |  | 0 | 0 |  | 4.0 | 1.4 |  | 0 | 0 |
| **12** | 50 | 0 | 0 |  | 2.0 | 6.7 |  | 0 | 0 |  | 0 | 0 |
| **13** | 50 | 0 | 0 |  | 4.0 | 5.8 |  | 0 | 0 |  | 0 | 0 |
| **14** | 50 | 0 | 0 |  | 6.0 | 18.2 |  | 14.0 | 11 |  | 0 | 0 |
| **15** | 50 | 0 | 0 |  | 2.0 | 7.7 |  | 10.0 | 57.6 |  | 0 | 0 |
| **16** | 50 | 0 | 0 |  | 4.0 | 5.3 |  | 8.0 | 15.8 |  | 0 | 0 |
| **17** | 50 | 0 | 0 |  | 14.0 | 197.3 |  | 8.0 | 15.8 |  | 0 | 0 |
| **18** | 50 | 0 | 0 |  | 0 | 0 |  | 14.0 | 3.4 |  | 2.0 | 0.5 |
| **19** | 50 | 0 | 0 |  | 0 | 0 |  | 0 | 0 |  | 0 | 0 |
| **20** | 50 | 2.0 | 0.5 |  | 0 | 0 |  | 0 | 0 |  | 0 | 0 |
| **21** | 50 | 4.0 | 1.9 |  | 2.0 | 0.5 |  | 0 | 0 |  | 0 | 0 |
| **22** | 50 | 36.0 | 35.5 |  | 4.0 | 1.9 |  | 0 | 0 |  | 0 | 0 |
| **23** | 50 | 24.0 | 10.6 |  | 8.0 | 18.2 |  | 0 | 0 |  | 2.0 | 0.5 |
| **24** | 50 | 22.0 | 17.3 |  | 0 | 0 |  | 2.0 | 0.5 |  | 0 | 0 |
| **25** | 50 | 14.0 | 6.7 |  | 0 | 0 |  | 2.0 | 5.8 |  | 0 | 0 |
| **26** | 50 | 26.0 | 11 |  | 0 | 0 |  | 6.0 | 1.4 |  | 0 | 0 |
| **27** | 50 | 0 | 0 |  | 4.0 | 193.4 |  | 0 | 0 |  | 0 | 0 |
| **28** | 50 | 0 | 0 |  | 0 | 0 |  | 0 | 0 |  | 0 | 0 |
| **29** | 50 | 0 | 0 |  | 0 | 0 |  | 0 | 0 |  | 0 | 0 |
| **30** | 50 | 0 | 0 |  | 50.0 | 106.1 |  | 0 | 0 |  | 4.0 | 5.8 |
| **31** | 50 | 0 | 0 |  | 0 | 0 |  | 0 | 0 |  | 0 | 0 |
| **32** | 50 | 0 | 0 |  | 2.0 | 45.1 |  | 0 | 0 |  | 0 | 0 |
| **33** | 50 | 0 | 0 |  | 18.0 | 1168.8 |  | 0 | 0 |  | 4.0 | 1.9 |
| **34** | 50 | 0 | 0 |  | 8.0 | 33.6 |  | 0 | 0 |  | 0 | 0 |
| **35** | 50 | 0 | 0 |  | 0 | 0 |  | 0 | 0 |  | 0 | 0 |
| **36** | 50 | 0 | 0 |  | 0 | 0 |  | 0 | 0 |  | 0 | 0 |
| **37** | 50 | 0 | 0 |  | 0 | 0 |  | 0 | 0 |  | 0 | 0 |
| **38** | 50 | 0 | 0 |  | 0 | 0 |  | 0 | 0 |  | 0 | 0 |
| **39** | 50 | 0 | 0 |  | 8.0 | 16.8 |  | 0 | 0 |  | 0 | 0 |
| **40** | 50 | 0 | 0 |  | 8.0 | 18.7 |  | 0 | 0 |  | 0 | 0 |
| **41** | 50 | 0 | 0 |  | 4.0 | 1.4 |  | 0 | 0 |  | 2.0 | 0.5 |
| **42** | 50 | 0 | 0 |  | 0 | 0 |  | 0 | 0 |  | 0 | 0 |
| **43** | 50 | 0 | 0 |  | 0 | 0 |  | 0 | 0 |  | 0 | 0 |
| **44** | 50 | 0 | 0 |  | 0 | 0 |  | 0 | 0 |  | 0 | 0 |
| **45** | 50 | 0 | 0 |  | 8.0 | 4.3 |  | 0 | 0 |  | 0 | 0 |
| **46** | 50 | 0 | 0 |  | 0 | 0 |  | 0 | 0 |  | 0 | 0 |
| **47** | 50 | 0 | 0 |  | 8.0 | 10.6 |  | 0 | 0 |  | 0 | 0 |
| **48** | 50 | 0 | 0 |  | 6.0 | 8.6 |  | 0 | 0 |  | 0 | 0 |
| **49** | 50 | 0 | 0 |  | 2.0 | 13 |  | 0 | 0 |  | 0 | 0 |
| **50** | 50 | 0 | 0 |  | 2.0 | 1.9 |  | 0 | 0 |  | 0 | 0 |
| **51** | 50 | 0 | 0 |  | 6.0 | 8.2 |  | 2.0 | 1 |  | 0 | 0 |
| **52** | 50 | 0 | 0 |  | 2.0 | 1.4 |  | 4.0 | 1 |  | 0 | 0 |
| **53** | 50 | 0 | 0 |  | 8.0 | 5.3 |  | 2.0 | 3.8 |  | 0 | 0 |
