# Supplementary material for: Comparison of individual and pooled diagnostic examination strategies during the national mapping of soil-transmitted helminths and Schistosoma mansoni in Ethiopia
Source: PLoS Negl Trop Dis. 2018 Sep 10;12(9):e0006723. doi: 10.1371/journal.pntd.0006723 (PMC6147605; doi:10.1371/journal.pntd.0006723)
Supplement: S3 Table — (DOCX) [file pntd.0006723.s007.docx]

Supplementary Table 3. The absolute and proportional changes in total operational costs when the costs varied by 10%.The accessibility of the schools was defined as poor, moderate and high.Schools are poorly accessible, when only on school per daycan be surveyed. Schools are moderately and highly accessible, when 2 and 3 schools per day can be surveyed, respectively. In each scenario of school accessibility, 50 subjects are randomly selected per school.

|  | | Individual examination strategy | | |  | Pooled examination strategy | | |  | | Cost-savings when a pooled examination strategy is applied (%) | | | |
| --- | --- | --- | --- | --- | --- | --- | --- | --- | --- | --- | --- | --- | --- | --- |
|  |  | Estimated operational costs (US$) | Absolute change in total operational costs when the costs varied by 10% (US$)^a^ | Proportional change in total operational costs when the costs varied by 10% (%)^a^ |  | Estimated operational costs (US$) | Absolute change in total operational costs when the costs varied by 10% (US$)^a^ | Absolute proportional change in total operational costs when the costs varied by 10% (%)^a^ |  | | Estimated cost savings (%) | | Absolute change in cost savings when the cost is reduced by 10% | Absolute change in cost savings when the cost is increased by 10% |
| Poor school accessibility | | | | | | | | | | | | | | |
|  | Material | 12,161.3 | +/- 18.0 | +/- 0.1 |  | 10,826.2 | +/- 1.8 | +/- 0.0 |  | | 11.0 | | 0.1 | -0.1 |
|  | Salary | 12,161.3 | +/- 460.3 | +/- 3.8 |  | 10,826.2 | +/- 345.2 | +/- 3.2 |  | | 11.0 | | 0.6 | -0.5 |
|  | Data entry | 12,161.3 | +/- 2.5 | +/- 0.0 |  | 10,826.2 | +/- 0.2 | +/- 0.0 |  | | 11.0 | | 0.0 | 0.0 |
|  | Fee teachers | 12,161.3 | +/- 64.8 | +/- 0.5 |  | 10,826.2 | +/- 64.8 | +/- 0.6 |  | | 11.0 | | -0.1 | 0.1 |
|  | Rent car | 12,161.3 | +/- 554.4 | +/- 4.6 |  | 10,826.2 | +/- 554.4 | +/- 5.1 |  | | 11.0 | | -0.5 | 0.5 |
|  | Fuel | 12,161.3 | +/- 116.1 | +/- 1.0 |  | 10,826.2 | +/- 116.1 | +/- 1.1 |  | | 11.0 | | -0.1 | 0.1 |
|  |  |  |  |  |  |  |  |  |  | |  | |  |  |
| Moderate school accessibility | | | | | | | | | | | | | | |
|  | Material | 12,801.0 | +/- 24.0 | +/- 0.2 |  | 11,404.5 | +/- 2.4 | +/- 0.0 | |  | | 10.9 | 0.1 | -0.1 |
|  | Salary | 12,801.0 | +/- 460.3 | +/- 3.6 |  | 11,404.5 | +/- 345.2 | +/- 3.0 | |  | | 10.9 | 0.5 | -0.5 |
|  | Data entry | 12,801.0 | +/- 3.3 | +/- 0.0 |  | 11,404.5 | +/- 0.3 | +/- 0.0 | |  | | 10.9 | 0.0 | 0.0 |
|  | Fee teachers | 12,801.0 | +/- 86.4 | +/- 0.7 |  | 11,404.5 | +/- 86.4 | +/- 0.8 | |  | | 10.9 | -0.1 | 0.1 |
|  | Rent car | 12,801.0 | +/- 554.4 | +/- 4.3 |  | 11,404.5 | +/- 554.4 | +/- 4.9 | |  | | 10.9 | -0.5 | 0.5 |
|  | Fuel | 12,801.0 | +/- 151.7 | 1.2 |  | 11,404.5 | +/- 151.7 | 1.3 | |  | | 10.9 | -0.1 | 0.1 |
|  |  |  |  |  |  |  |  |  | |  | |  |  |  |
| High school accessibility | | | | | | | | | | | | | | |
|  | Material | 13,590.8 | +/- 32.0 | +/- 0.2 |  | 12,112.4 | +/- 3.2 | +/- 0.0 | |  | | 10.9 | 0.2 | -0.2 |
|  | Salary | 13,590.8 | +/- 460.3 | +/- 3.4 |  | 12,112.4 | +/- 345.2 | +/- 2.9 | |  | | 10.9 | 0.5 | -0.5 |
|  | Data entry | 13,590.8 | +/- 4.4 | +/- 0.0 |  | 12,112.4 | +/- 0.4 | +/- 0.0 | |  | | 10.9 | 0.0 | 0.0 |
|  | Fee teachers | 13,590.8 | +/- 115.2 | +/- 0.8 |  | 12,112.4 | +/- 115.2 | +/- 1.0 | |  | | 10.9 | -0.1 | 0.1 |
|  | Rent car | 13,590.8 | +/- 554.4 | +/- 4.1 |  | 12,112.4 | +/- 554.4 | +/- 4.6 | |  | | 10.9 | -0.5 | 0.4 |
|  | Fuel | 13590.8 | +/- 192.8 | +/- 1.4 |  | 12,112.4 | +/- 192.8 | +/- 1.6 | |  | | 10.9 | -0.2 | 0.2 |

a: a positive value corresponds with a decrease of the costs with 10%, a negative value with an increase in costs with 10%
